# Choice of fluids in critically ill patients

**DOI:** 10.1186/s12871-018-0669-3

**Published:** 2018-12-22

**Authors:** Claude Martin, Andrea Cortegiani, Cesare Gregoretti, Ignacio Martin-Loeches, Carole Ichai, Marc Leone, Gernot Marx, Sharon Einav

**Affiliations:** 1 Department of Anesthesia, Intensive Care and Trauma Center, Nord University Hospital, Aix Marseille University, APHM, Marseille, France; 2 Department of Surgical, Oncological and Oral Science (Di.Chir.On.S.). Section of Anesthesia, Analgesia, Intensive Care and Emergency. Policlinico Paolo Giaccone. University of Palermo, Via del vespro 129, 90127 Palermo, Italy; 3 St Jame’s hospital and Trinity College Dublin, Dublin, Ireland; 4 Universidad de Barcelona. CIBER, Barcelona, Spain; 5 Adult Intensive Care Unit, Université Côte d’Azur, University Medicine of Nice, Nice, France; 6 Department of Anesthesiology and Critical Care Medicine, Aix Marseille University, Assistance Publique Hopitaux de Marseille, Marseille, France; 7 Department of Intensive Care Medicine, University Hospital RWTH Aachen, Aachen, Germany; 8 Surgical Intensive Care Unit, Shaare Zedek Medical Centre, Jerusalem, Israel; 9 Hebrew University Faculty of Medicine, Jerusalem, Israel

**Keywords:** Fluids, Resuscitation, Critically ill, Crystalloid, Colloid, Intensive care unit

## Abstract

**Background:**

Fluids are by far the most commonly administered intravenous treatment in patient care. During critical illness, fluids are widely administered to maintain or increase cardiac output, thereby relieving overt tissue hypoperfusion and hypoxia.

**Main text:**

Until recently, because of their excellent safety profile, fluids were not considered “medications”. However, it is now understood that intravenous fluid should be viewed as drugs. They affect the cardiovascular, renal, gastrointestinal and immune systems. Fluid administration should therefore always be accompanied by careful consideration of the risk/benefit ratio, not only of the additional volume being administered but also of the effect of its composition on the physiology of the patient. Apart from the need to constantly assess fluid responsiveness, it is also important to periodically reconsider the type of fluid being administered and the evidence regarding the relationship between specific disease states and different fluid solutions.

**Conclusions:**

The current review presents the state of the art regarding fluid solutions and presents the existing evidence on routine fluid management of critically ill patients in specific clinical settings (sepsis, Adult Respiratory Distress Syndrome, major abdominal surgery, acute kidney injury and trauma).

**Electronic supplementary material:**

The online version of this article (10.1186/s12871-018-0669-3) contains supplementary material, which is available to authorized users.

## Background

Fluids are probably the most commonly administered intravenous treatment in inpatient care. Because of their excellent safety profile, until recently fluid solutions were not considered “medications” [1]. Little to no thought was therefore invested in the choice of fluids to be administered in specific clinical scenarios. However, recent evidence on long-term effects has altered our view on the different types of fluids available for fluid resuscitation. Intravenous fluids should be seen as drugs affecting the cardiovascular, renal, gastrointestinal and immune systems and should therefore not be administered “blindly”.

Emphasis on the importance of volume above all the other characteristics of the fluids administered was nurtured by early guidelines that focused on administering specific fluid volumes to hemodynamically unstable patients (i.e. the surviving sepsis campaign) [2, 3]. It is true that fluid administration is an important component of treatment of overt tissue hypoperfusion and hypoxia. Fluids may expand the intra-vascular compartment, thereby improving cardiac output (CO) and end-organ perfusion [3, 4]. However, the most common error with regards to fluid administration is the belief that resuscitation hinges on transfusion of a specific volume of fluids [3, 5].

Disease processes are dynamic and their response to fluid may change over time. Specific disease states may also require different fluid therapy. Evidence from perioperative settings has associated both hypo- and hypervolemia with several unfavorable outcomes, including acute kidney injury (AKI), respiratory complications, increased lengths of stays, admission costs and 30-day-mortality rates [6, 7]. Later iterations of the guidelines have therefore clarified that the aim of fluid resuscitation is restoration of end-organ perfusion and correction of physiological imbalance. Follow-up during fluid administration should therefore include surrogate markers of organ perfusion (e.g. mean arterial pressure, central venous oxygen saturation, lactate, CO), markers of circulation, blood electrolyte and acid-base composition and indicators of renal function [3, 8]. No fluid is ideal for all disease conditions at all times. This review presents the current state of knowledge regarding the types of fluids to be administered with an emphasis on several disease states.

## Methods

The concept of this review was put forward during Euroanesthesia 2015, in the Intensive Care Subcommittee meeting which is open to all attendees. The subcommittee meeting is typically attended by intensive care physicians who are also anaesthesiologists with an interest in promoting research in their field. Following group discussion of several options proposed, the attending subcommittee members selected this topic as worthy of address. The authors to be approached were determined based on their previous contribution to the international literature on specific related topics and their writing experience. All those approached agreed to contribute.

For the first section of this paper (“Types of fluid”) a non-systematic search of Pubmed was performed. For the second part (“Fluid administration in specific disease conditions”) the services of a professional librarian were employed and a systematic search of the literature was performed. The systematic search was conducted in both Pubmed™ and Embase™ databases and included all publications until June 30th 2018. The Cochrane database is embedded in full in both of these databases therefore a separate search was not conducted for the Cochrane database. The key words used were “fluid administration” OR “fluid therapy” OR “fluid resuscitation” AND “ICU” OR “critically ill” OR trauma OR sepsis OR “major abdominal surgery” OR “respiratory distress syndrome” OR “acute kidney injury”. The filters applied included human subjects, adults and publication in the English language. Only studies with original data (observational, retrospective or prospective), reviews, systematic reviews and meta-analyses were included. After exclusion of duplicate publications this search yielded 3364 potential papers of interest (see Additional file 1).

**Fig. 1:**
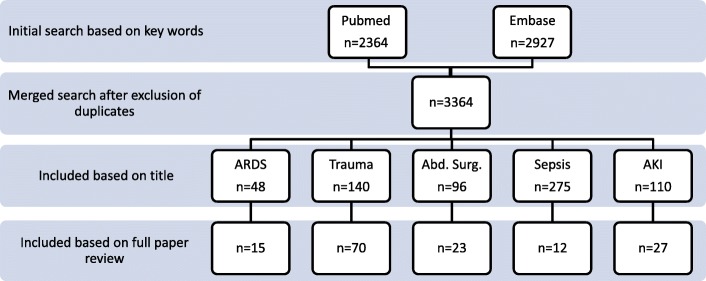
Flow diagram of the systematic search of the literature

The titles of this list of articles were screened five times by the authors. Each searched for papers with content relevant to their specific clinical condition of interest. Screening for sepsis was conducted by CDM and IML, for major abdominal surgery by SE, for acute respiratory distress syndrome (ARDS) by CG and AC, for trauma by ML, and for acute kidney injury by CI. Based on this initial screening 669 papers were selected for review of the abstract. For each section two of the authors then reviewed the abstracts and selected the articles for full download. Overall 147 articles were reviewed in full text (See Fig. 1 for the full publication inclusion/exclusion process). The references of these articles were then manually screened for additional potentially relevant papers. The two main selection criteria to determine final inclusion were relevance to the topic at hand and the quality of the paper based on expert opinion. For specific issues, additional seminal studies were used at the discretion of the authors.

### Types of fluids

The following section discusses the characteristics of most existing fluid solutions. The chemical composition of many of the solutions currently on the market is presented in Table 1.

**Table 1:** The chemical composition of commonly used intravenous fluid solutions

Solutions	Na^+^ (meq/L)	K^+^ (meq/L)	Cl^−^ (meq/L)	Other anions (meq/L)	Osmolarity (mosm/L)	In vivo SID^a^(meq/L)
Crystalloids						
Unbalanced						
NaCl 0.9%	154	0	154	–	308	–
NaCl 3%	510	0	510	–	1026	–
NaCl 7.5%	1275	0	1275	–	2395	–
Balanced						
Lactate Ringer	130	4	108	Lactate (27.6)	277	27
Acetate Ringer	132	4	110	Acetate (29)	277	27
Acetate Gluconate (Plasmalyte®)	140	5	98	Acetate (27)	294	50
				Gluconate (23)		
Acetate Malate (Isofundin®)	145	4	127	Acetate (24)	304	27
				Malate (5)		
Colloids						
Unbalanced						
Hydroxyethylstarch (Voluven®)	154	0	154	–	308	–
Albumin	154	0	154	–	308	–
Balanced						
Hydroxyethylstarch (Tetraspan®)	140	4	118	Acetate (24)	297	29
				Malate (5)		
Hydroxyethylstarch (Hextend®)	143	3	124	–	307	28
Gelatins 4% (Plasmion®)	154	0	120	–	307	32
Gelatins 3% (Gelofusin®)	150	0	100	–	284	56

^a^Strong Ion Difference

#### Crystalloids

Given the current controversy surrounding administration of colloids, crystalloids have prudently been selected as the first choice for fluid resuscitation. Unbalanced crystalloid solutions (i.e. saline solutions) typically contain high concentrations of sodium-chloride and have a pH that is lower than 6.0. In this sense, the term “normal” saline is a misnomer. The characteristics of saline solutions depend on their salt concentration (0.9, 0.45, 3% etc.). Balanced crystalloid solutions (e.g. Ringer’s lactate, Plasma-Lyte, Isofundine) are buffered by anions other than chloride. The chloride concentrations of balanced solutions therefore more closely approximate plasma but their osmolality is lower and they contain alternative anions in non-physiological concentrations. Lactate-buffered fluids are the least costly in this fluid category.

##### Crystalloids, chloride concentrations and renal failure

The concentration of chloride in 0.9% saline solution exceeds that of plasma (154 mEq/L). Experimental studies have shown that high renal tubular chloride concentrations induce renal afferent vasoconstriction with a resultant decrease in renal blood flow and GFR [9, 10]. No similar effect has been observed with relation to elevated sodium concentrations [11]. Moreover, canine models demonstrate that when accompanied by hypovolemia, the reduction in renal blood flow doubles compared to euvolemia [11]. In humans, administration of isotonic saline has been shown to cause hyperchloremic acidosis in both non critically ill [12] and critically ill patients [13, 14]. In healthy human volunteers, administration of intravenous 0.9% saline has also been shown to decrease renal blood flow velocity and renal cortical tissue perfusion when compared to a balanced solution (e.g. plasma-lyte 148) [15].

**Summary statements:**

- Animal and human studies demonstrate that high renal tubular chloride concentrations induce renal afferent vasoconstriction with a resultant decrease in renal blood flow.
- Given that the availability and cost of saline and balanced crystalloids are not significantly different, saline should probably no longer be used for intravascular volume expansion.

### Colloids

Colloids contain macromolecules such as hydroxyethyl-starch (HES), gelatin, dextran, or albumin. In the past colloids were thought to be distributed primarily in the intravascular space and were therefore considered 3–4 times more effective than crystalloids for restoring intravascular volume. Clinical evidence supports the assumption of higher intravascular retention of colloids, albeit not to such extent. Administration of 1400–1800 ml of gelatin, albumin, and HES increases cardiac index by 25–44% in surgical patients while administration of the same amount of saline (1800 ml) does not affect cardiac index [16]. Clinical hemodynamic stabilization also seems to occur more rapidly and with smaller volumes of colloids compared to crystalloids [17]. Unfortunately, many studies yielding such evidence were not designed for this purpose, which limits the validity of their findings.

Today it is clear that the ratio of intravascular to administered volume of colloids is usually only 1:1.2 [4, 16–19], far less than previously believed. Large multicentre, randomised trials have shown ratios < 1:2 [16–19]. Furthermore, many trials noting decreased transfusion requirements with the use of colloids are being criticised for bias, as fluid therapy was often determined by the treating clinicians [17–19].

#### Hetastarch (HES)

Three large RCTs have associated administration of HES with AKI and the need for RRT in ICU patients, especially in those with sepsis [19]. Three randomized controlled studies comparing intraoperative administration of HES versus crystalloids yielded conflicting results; HES was responsible for an increased incidence of renal dysfunction in two studies [20, 21] but no such effect was observed in the third [22].

The findings from meta-analyses suggest this finding may depend on the patient cohort. Three meta-analyses (two including general critically ill patients and one septic patients receiving fluids for resuscitation) confirmed the higher risk of AKI but reported conflicting results for mortality [23–25]. One further meta-analysis comparing HES to crystalloids in RCTs of patients without sepsis did not demonstrate any difference in the incidence of RRT or overall mortality. In this analysis, however, the total volume of fluids administered to patients receiving colloids was lower [26] raising questions regarding the parallel protective effect of administration of less fluids. Two meta-analyses performed in surgical patients showed that intraoperative HES administration did not increase either the incidence of AKI or mortality [27, 28].

Nonetheless in 2013, The European Medicines Agency decided that HES should not be used in critically ill patients in the EU and the US due to lack of supportive evidence and some safety concerns [29, 30]. More recently, the Co-ordination group for Mutual recognition and Decentralised procedures – human (CMDh) of the European Medicines Agency (EMA) recommended suspension of marketing authorisations for HES (apart from controlled clinical trials), “because of the risk of kidney injury and death in certain patient populations” [29].

#### Albumin

Albumin is the only natural colloid used for intravascular volume replacement in humans. In the past, administration of albumin was thought to increase mortality. However, in 2013, a repeat Cochrane meta-analysis found no evidence of such adverse effect [31]. The multicentre Saline versus Albumin Fluid Evaluation (SAFE) study performed in 2004 was probably the decisive factor in this reappraisal. In the SAFE study, no difference was found between hypovolaemic patients treated with albumin (*n* = 3497) or saline (*n* = 3500) in mortality, length of ICU or hospital stay, or organ dysfunction [18]. The main criticisms of the SAFE study are that the presence of hypovolemia was not determined based on predetermined criteria and that the dose of fluid to be administered was not preset [18].

Three meta-analyses have studied whether human albumin affects mortality when administered for intravascular volume expansion to critically ill patients with sepsis [32–34]. These are discussed in greater detail in the section on sepsis (see below). Taken together, it is safe to state there is no good-quality evidence regarding the value of resuscitation of critically ill patients using albumin.

**Summary statements:**

- It remains unclear whether albumin confers either benefit or risk in terms of mortality and renal function.
- Given the cost of human albumin, it should generally not be considered the first choice for fluid replacement unless there is a specific indication for its use.

#### Gelatin

Gelatin is a synthetic colloid with a molecular weight of ~ 35 kDa and a relatively short plasma half-life (approximating 2-3 h). The recent debate on colloids has focused on the adverse effects of gelatin; namely increased renal injury, coagulopathy, anaphylaxis and mortality. Unfortunately few studies on gelatin have been sufficiently powered to reveal valid patient-centered outcomes [31, 35, 36]. Adequately powered controlled, randomised, double-blinded trials, such as GENIUS-trial which is currently recruiting (NCT02715466) are required.

Meta-analyses studying potential unwanted effects of gelatin (predominantly compared to crystalloids) have not shown increased renal injury, clinically relevant bleeding [36, 37] or even mortality [31, 35, 36, 38]. Bayer et al. used a sequential design to study three regimens of fluid administration to ICU patients [39]; HES plus crystalloids, Gelatin plus crystalloids, and crystalloids alone. The rate of renal replacement therapy was lower with crystalloids alone. Mortality, blood transfusion, and allergies did not differ [39, 40]. Despite the limitations of this study (i.e. confounding by inconsistent reporting and time-related treatment changes and differences in the volume of priming) these have been supported by Moeller et al. who report that German pharmacovigilance data do not indicate gelatin-induced renal injury [36]. Corroboration can also be found in a recent systematic review which reported a decreased risk of renal failure with gelatin when compared to any other intravenous fluid [41].

With regards to allergic reactions, one meta-analysis reported a significantly greater incidence of allergic responses with gelatin compared to crystalloids or albumin [36]. This result was dominated by a single study where urea-linked gelatine was used [42]. Urea-linked gelatine is far more allergenic than modified fluid gelatine (MFG), which exists in most such solutions to-date [43]. Allergic reactions to gelatin are typically mild and their incidence is much lower when MFG is used compared to older gelatin preparations [42, 43]. Existing evidence does not clearly demonstrate that gelatine has more adverse effects. However, the evidence on this topic remains clearly insufficient.

**Summary statements:**

- The evidence on gelatins remains clearly insufficient; few RCTs have been sufficiently powered to reveal valid patient-centered outcomes.
- Observational studies in large cohorts and meta-analyses comparing gelatine to crystalloids have mostly shown no different or even lower rates of renal injury, clinically relevant bleeding and death with gelatins.
- Allergic reactions are more common and more severe with urea-linked gelatin than with modified fluid gelatine but most studies comparing gelatins to crystalloids/albumin have failed to differentiate between the two.

### Dextrans

The CMDh statement suggests that dextrans may be used as alternative fluid solutions in routine clinical practice [29]. This recommendation is somewhat questionable given the paucity of data regarding dextrans to-date.

Early trials studying EGDT either used no colloids at all [44] or were not explicit regarding the specific fluid solutions used [45–47]. The 6S [19], VISEP [17], CHEST [4], and CRYSTMAS [16] studies included no fluid solutions that contain dextrans. In the CRISTAL trial only five of the 1414 patients receiving colloids were treated with solutions containing dextrans [48]. A search of PubMed using the keywords “dextran/TI AND volume/AB” (1998 to January 2018) yielded only 17 studies that describe the use of dextrans in humans and most of these were small studies focusing on dextran-based hyperoncotic therapy.

**Summary statement:**

- The CMDh stated that there are no legal constraints regarding the use of dextrans for intravascular fluid replacement at this time. Although this statement is probably true, there is also an alarming lack of evidence to support the recommendation to use these solutions in critically ill humans.

### In summary

Balanced crystalloids are generally the solutions of choice for intravascular fluid resuscitation of hypovolaemic patients. The evidence against isotonic saline remains inconclusive but the possible risks associated with its use are not balanced by any advantage in therapeutic efficacy or cost at this time. Colloids are probably more effective than crystalloids, albeit not as effective as theoretically expected. However, no additional benefit has been conclusively proven for colloids over crystalloids. Therefore, if a decision has been made to administer colloids, it should always follow crystalloid administration. The solutions to consider after failed treatment with crystalloids should be either albumin or MFG. Regardless of the choice of colloids to be used, colloid administration should be considered rescue therapy and remain limited to profound, acute hypovolaemia.

### Fluid administration in specific disease conditions

As noted above, there is accumulating evidence that specific disease states may require different fluid therapy. In the section below the data supporting this statement is presented for specific disease conditions often seen in the ICU (i.e. sepsis, major abdominal surgery, ARDS trauma and AKI). The guidelines published regarding fluid administration in these disease conditions are summarised in Table 2. Additional file 2 reports some of the most relevant articles retrieved by the systematic search.

**Table 2:** Guidelines on fluid management and resuscitation

Guideline title	Authors, year	Recommendations	Grade
Surviving Sepsis Campaign: international guidelines for management of sepsis and septic shock: 2016	Rhodes A. et al. 2017 [2]	We recommend that a fluid challenge technique be applied where fluid administration is continued as long as hemodynamic factors continue to improve	Best practice statement
		We recommend crystalloids as the fluid of choice for initial resuscitation and subsequent intravascular volume replacement in patients with sepsis and septic shock	1B
		We suggest using either balanced crystalloids or saline for fluid resuscitation of patients with sepsis or septic shock	2C
		We suggest using albumin in addition to crystalloids for initial resuscitation and subsequent intravascular volume replacement in patients with sepsis and septic shock when patients require substantial amounts of crystalloids.	2C
		We recommend against using hydroxyethyl starches (HESs) for intravascular volume replacement in patients with sepsis or septic shock	1A
		We suggest using crystalloids over gelatins when resuscitating patients with sepsis or septic shock	2C
The clinical practice guideline for the management of ARDS in Japan	Hashimoto et al. 2017 [64]	We suggest fluid restriction in the management of adult patients with ARDS.	2B Weak recommendation Moderate quality evidence
Scandinavian clinical practice guidelines in fluid and drug therapy in adults with acure respiratory distress syndrome	Claesson et al. 2016 [65]	We suggest fluid restriction over a liberal fluid strategy in adults with ARDS	Weak recommendation Moderate quality evidence
European guideline on management of major bleeding and coagulopathy following trauma	Rossaint et al., 2016 [88]	We recommend that fluid therapy using isotonic crystalloid solutions be initiated in the hypotensive bleeding trauma patient	1A
		We suggest that excessive use of 0.9% NaCl solution be avoided	2C
		We recommend that hypotonic solutions such as Ringer’s lactate be avoided in patients with severe head trauma	1C
		We suggest the use of colloids be restricted due to the adverse effects on haemostasis	2C
AKI in the perioperative period & in ICU: french expert recommendations	Ichai C et al. 2016 [111]	We recommend not administering hydroxyethylstarch (HES) in the ICU. STRONG agreement	1B
		We suggest the preferential use of crystralloid instead of colloid for fluid loading. STRONG agreement	2A
		We suggest preferring balanced solutions in case of large volume loading. STRONG agreement	2A
		After hemodynamic stabilisation, we suggest avoiding fluid overload in the ICU. STRONG agreement	2A

The table also reports the strenght of recomemantions and GRADE

### Sepsis

Lactic acidosis is a major metabolic side effect of sepsis. As noted above, intravenous administration of 0.9% saline may cause iatrogenic hyperchloremic acidosis [12, 49]. Hyperchloremia has been associated with increase in mortality in both septic and non-septic patients [50]. However, most studies examining this issue were retrospective, which precludes derivation of a meaningful causative association between the two. Studies comparing solutions with high versus low-chloride concentrations have yielded conflicting results thus far. Reduced rates of mortality and AKI have been described with balanced solutions [12, 13, 15, 49, 51] therefore until more information from RCTs is available, balanced solutions remain preferred over 0.9% saline for the treatment of hemodynamically unstable septic patients.

#### Albumin

Albumin is the main determinant of plasma oncotic pressure and has a pivotal role in regulating fluid dynamics at the microvascular level. Albumin also performs other functions that may be relevant for septic patients. These include stabilization of the glycocalyx, transport of molecules, antioxidant effects, immuno-modulation and positive inotropic effects.

In the SAFE trial, patients admitted to the ICU were randomly assigned to receive albumin or 0.9% saline for intravascular-fluid resuscitation for 28 days and no difference was observed in all-cause mortality. However, the subgroup analysis of septic patients (planned a-priori) showed an adjusted odds ratio for death of 0.71 (95% CI: 0.52, 0.97, *p* = 0.03) for albumin [18].

The ALBIOS trial, which compared administration of albumin (target plasma concentration of 30 g/L) to crystalloids alone showed no difference in outcomes in the study population as a whole and in the subgroups of patients with severe sepsis and septic shock [52]. However, patients with septic shock who were randomised to receive albumin had higher 90-day survival rates (6.3% *p* = 0.04) [52].

As noted above, three meta-analyses have recently studied whether human albumin affects mortality when administered for intravascular volume expansion to critically ill patients with sepsis [32–34]. Two of these studies included patients who received crystalloids as well as synthetic colloids in the control arm. The mortality rates were equivalent in the two groups in both of these studies [32, 33]. The third meta-analyses was performed using only crystalloids as the comparator and did not include the data from the EARSS trial which was available only as an abstract [53]. In this meta-analysis, the 90-day mortality of patients in septic shock was significantly lower with albumin [34]. This is concordant with another meta-analysis performed in patients with septic shock [54].

**Summary statements:**

- Much of data available regarding the type of fluid to be preferred in patients with sepsis and/or septic shock comes from subgroup or meta-analyses.
- The data suggests that albumin may reduce morbidity and survival in patients with septic shock.
- As a rule, volume substitution septic patients should be undertaken using crystalloids, probably balanced solutions.
- HES must not be used in critically ill patients, septic or not.
- If acute hypovolaemia is not responsive to crystalloids alone, the use of human albumin can be considered.

### Adult respiratory distress syndrome

ARDS was initially considered an inflammatory protein-rich pulmonary edema accompanied by leakage of protein-rich fluids into the interstitial space. The resultant increase in lung weight was thought to generate atelectasis with eventual impairment of lung mechanics and gas exchange [55, 56]. However, ARDS has both inflammatory edema and hydrostatic components [55, 57, 58]. Development of pulmonary hypertension may lead to an increase in hydrostatic pressure [55]. Activation of the renal aldosterone-angiotensin system during mechanical ventilation also generates high increased intrathoracic pressure which causes water and salt retention [59, 60]. Fluid loading may improve hemodynamics and oxygenation but it may also worsen lung aeration in patients with lung inflammation through several mechanisms [61]. Moreover, a positive fluid balance in patients with ARDS may increase mortality rate [62].

Data about the best type of fluid in patients with ARDS are scarce. A recent meta-analysis investigated the effect of colloids versus crystalloids in patients with ARDS. Three trials were included for a total of 206 patients. All the included studies compared albumin versus saline. The meta-analysis found improved oxygenation but no survival benefit in patients treated with albumin versus crystalloid [63]. However, the risk of bias of included trials ranged from unclear to high and the sample size was very low.

**Summary statements:**

- Fluid management of patients with ARDS has significantly improved over the last two decades but many aspects require clarification.
- Conservative strategies seem to lead to better oxygenation and shorter periods of mechanical ventilation. Although the evidence supporting it is still of moderate quality, conservative fluid administration is recommended in patients with ARDS [64, 65].
- The type, timing and dose of fluids to be administered must still be evaluated per-case [53], taking into account the etiology of ARDS (e.g. burns, TBI, infection), patient comorbidities and hemodynamic and respiratory condition [66]
- The type of monitoring used is less important than the composition of the fluids administered and overall fluid balance [67–69].

### Major abdominal surgery

Fluid administration is part of the perioperative routine in both elective and urgent major abdominal surgery but these two situations could not differ more. Elective major abdominal surgery is often accompanied by bowel preparation [70–72], preoperative cardiac assessment when indicated and is performed on a patient that is hemodynamically stable and adequately hydrated. Conversely, patients undergoing urgent abdominal surgery often suffer severe intravascular fluid depletion due to both intestinal and extra-intestinal losses (e.g. vomiting, extra-vascular leakage), are often hemodynamically unstable, and have usually undergone little preoperative assessment.

*Elective surgery* - The sparse literature addressing perioperative fluid administration in patients undergoing major abdominal surgery refers to elective patients [73]. Although mechanical bowel preparation is no longer recommended [74], many patients still undergo drug-induced bowel preparation. Similarly the evidence-based recommendation to allow ingestion of clear fluids up to 2 h before surgery is often translated to fasting from midnight on the day before surgery [75]. Such practice may induce dehydration and electrolyte imbalance despite institution of corrective hydration.

In this clinical scenario, intraoperative hydration is generally titrated to cover the fluid deficit resulting from bowel preparation and fasting as well as routine fluid maintenance (2–3 ml/kg/h). With adequate preoperative preparation however, the fluid deficit in these patients rarely exceeds 2.5% of body weight. Yet, traditional rehydration during surgery has been shown to result in administration of 7 l of fluid on the day of surgery and a weight gain of 3–6 kg [76–78]. Such practice has led to the current speculation regarding the impact of perioperative fluid administration (both volume and type) on patient physiology.

One ongoing treatment dilemma is whether adding vasopressor therapy to fluid administration is beneficial since such practice may decrease the amount of fluid administered. An early meta-analysis of intra-operative hemodynamic optimization achieved by combining fluids and vasopressors compared to fluids alone showed a decrease in both renal and gastrointestinal complications, but later multicentre trials have yielded mainly controversial results [79–81]. Most of these studies follow patients either throughout admission or to 28 days after surgery. However, none present any data regarding post-operative fluid management, which may have determined the outcomes sought during this time frame.

Regarding the choice of fluids, most discussion still surrounds the issue of crystalloids versus colloids [82]. While newer data does not suffice as yet to support the use of colloids, neither does it suggest that risk is increased. Conversely, there is some evidence that gastro-intestinal outcomes may even be slightly better with colloids [83]. This finding is supported by animal studies suggesting that goal-directed colloid fluid therapy increases microcirculatory blood flow and tissue oxygen tension in healthy and injured peri-anastomotic colon compared to goal-directed or restricted crystalloid fluid therapy [84]. With regards to a direct comparison between balanced crystalloid solutions versus normal saline, even less literature exists. An RCT comparing these solutions in major abdominal surgery demonstrated that balanced solutions caused less electrolyte disturbances, acid-base disequilibrium and increases in NGAL levels and were associated with a stronger anti-inflammatory effect [85].

*Urgent surgery* - Patients undergoing urgent abdominal surgery often present with sepsis or septic shock. Therefore, the principles guiding fluid administration in sepsis should also guide perioperative fluid administration. An average patient with a hollow viscus perforation who presents to the department of emergency medicine is likely to receive at least 1–2 l of crystalloids before surgery and several litres more during induction of anaesthesia and throughout surgery. These should not be discounted when initiating fluid therapy in the ICU after surgery. The choice of fluids to be administered should be determined by timely information regarding acid-base and electrolyte balance with particular emphasis on avoidance of an unnecessary chloride load. In the setting of severe extravascular leakage, intravascular fluid repletion with crystalloids alone may decrease tissue capillary density, thereby worsening microcirculatory flow dynamics and oxygen delivery. An overload of crystalloid solution may decrease oncotic pressure and viscosity and exacerbate the inflammatory response [86]. Hence the importance of considering the type of fluid in further resuscitation.

**Summary statements:**

- Adequate preoperative preparation for elective major abdominal surgery should not induce a fluid deficit exceeding 2.5% of body weight.
- Most studies regarding fluid administration in the perioperative setting are limited to early therapy.
- Intraoperative/postoperative rehydration of elective cases should be performed with a balanced salt solution. Although this may be accompanied by an increase in circulating cytokines no clinically deleterious effect has been observed.
- Colloids may be administered in elective surgery cases if required- there is no evidence of increased risk in this patient population and there is evidence of better gastrointestinal microcirculatory blood flow and tissue oxygen tension.
- Adding vasopressor therapy to fluid administration remains controversial - while it likely decreases the amount of fluid administered it may also decrease end organ perfusion.
- The principles guiding fluid administration in sepsis should also guide perioperative fluid administration in patients undergoing urgent abdominal surgery.
- The crystalloid chosen for patients after urgent abdominal surgery should be determined individually, based on patient condition at the time of ICU arrival.

### Trauma

Recent years have seen some interesting changes in fluid management of trauma patients. Although severe bleeding is the lead cause of death in trauma patients [87], the European guidelines for management of major bleeding and coagulopathy following trauma strongly recommend restricting volume replacement during initial trauma resuscitation [88]. This recommendation is based on data showing not only the feasibility of this approach but also its advantages in term of both process (e.g. hospital length of stay) and outcomes (e.g. survival) [89, 90].

For many years treatment with colloids was considered particularly efficacious in trauma patients. This concept was based on the assumption that the vascular endothelium remains intact after trauma (contrary to septic shock) [91]. Early experimental data supported this assumption, showing that resuscitation with HES 130/0.4 was superior to lactated Ringer [92]. In humans, an exploratory study of patients monitored with a pulmonary artery catheter showed similar hemodynamic outcomes with a lower volume of colloids than crystalloids [93]. However, subgroup analyses of trauma patients included in the RCTs comparing colloids and crystalloids have since failed to confirm this assumption with regards to wither mortality [48] or transfusion requirements [94]. In patients with TBI, mortality was actually higher with albumin than with saline, probably due to the greater increase in intracranial pressure observed during administration of albumin [95]. The European guidelines for management of major bleeding and coagulopathy following trauma therefore recommend isotonic crystalloids rather than colloids for initial resuscitation of hypotensive bleeding trauma patients [88].

Among crystalloid solutions, the respective roles of balanced solutions and saline remain controversial. Unsurprisingly, administration of lactated Ringer solution increases plasma lactate concentrations, whereas normal saline increases the base deficit [96]. In patients with severe TBI, hypotonic solutions (including lactated Ringer) should be avoided as they exacerbate cerebral edema. Conversely, balanced solutions cause less hyperchloremic acidosis than saline in these patients [97]. A RCT of adult trauma patients requiring blood transfusion, intubation, or operation within 60 min of arrival showed that pre-hospital resuscitation with Plasma-Lyte A yielded better acid-base status and less hyperchloremia 24-h after injury compared with saline [98]. To summarise - the use of balanced solutions seems promising for trauma resuscitation but currently remains under investigation [99].

There is ongoing debate regarding intravascular volume expansion with hypertonic saline in trauma patients [100, 101]. Han et al. randomized 294 patients with hypovolemic shock after trauma to receive 3% hypertonic saline (*n* = 82), 7.5% hypertonic saline (*n* = 80), or lactated Ringer (*n* = 84) [102]. Although baseline population characteristics were similar in the three groups, patients receiving hypertonic solutions (3% or 7.5%) were given about half the amount of fluids than those given lactated Ringer within the first hour, a difference which disappeared within 24 h. Some side effects (e.g. arrhythmia, hypernatremia) were more commonly observed in patients receiving 7.5% hypertonic saline, whereas others (e.g. renal failure, coagulopathy, pulmonary edema) were more prevalent among patients receiving lactated Ringer. The authors concluded that among the solutions examined 3% hypertonic saline has the best safety and efficacy profile [102]. With regards to colloids - the relative contribution of micro-circulatory abnormalities, endothelial dysfunction, local and systemic inflammatory processes and oxidative stress differs between hemorrhagic and septic shock. Decreased tissue perfusion is a major component of haemorrhagic shock whereas inflammatory processes are likely more predominant in septic shock. Hence the effects of HES may also differ. Evidence supporting the presence of a difference includes three meta-analysis showing that the use of HES was not associated with renal effects or clinically significant coagulopathy in the OR [27, 28, 103]. Similarly, no study found deleterious effects of HES in early resuscitation of trauma patients [104]. The European Medicine Agency decided that HES can still be used in surgical patients, and for management of hemorrhagic shock following an initial fluid challenge with crystalloids that has failed. However the clinician should be aware that colloids have not been associated with an improvement in survival in patients with trauma, burns or following surgery [31].

**Summary statements:**

- In the hypotensive trauma patient, crystalloids should be administered initially and the amount of fluids administered should be restricted.
- Colloids and hypertonic solutions may accelerate achievement of hemodynamic goals, but have been associated with clinically important side effects and have not been shown to decrease mortality. Therefore these solutions should not be used as first line therapy.
- Albumin and hypotonic saline should not be administered to patients with TBI.
- The debate between balanced crystalloids and normal saline in trauma remains open, but balanced crystalloids are preferred for large volume resuscitation.

### Acute kidney injury

Fluid administration is one of the cornerstones of prevention of AKI. As with any other body organ, the goal of fluid therapy in this clinical scenario is restoration of intravascular volume with secondary improvement in kidney perfusion pressures and a resultant improvement in local tissue oxygenation. However, the precise relationship between hypo/hypervolemia and AKI remains unclear. Studies differ substantially in case mix, fluid volumes and types and the timing of fluid administration. Regardless of the cause and/or mechanism of AKI, macro-circulation alterations (i.e. changes in renal blood flow) are associated with micro-circulation abnormalities (tissue perfusion), endothelial dysfunction, local and systemic inflammatory processes and oxidative stress [105]. The relative contribution of each of these to the development of AKI differs dependent on the cause of renal injury [105]. Whereas decreased tissue perfusion is a major component of haemorrhagic shock, inflammatory processes may be more predominant in AKI caused by septic shock [105]. Patients with sepsis seem particularly susceptible to the deleterious effects of hypervolemia on kidney function [106]. The importance of microcirculatory changes in this clinical scenario makes the choice of fluids all the more crucial.

#### Gelatins and albumin

Few studies have assessed the potential renal toxicity of gelatins [36, 38, 107]. An RCT comparing gelatins and crystalloids for fluid resuscitation in septic patients is currently ongoing (NCT 02715466). The RARE trial compared albumin to cystalloids in ICU patients and failed to demonstrate any increase in the risk of AKI [52].

**Summary statements:**

- Administration of HES increases the incidence of AKI and RRT in critically ill patients. The use of HES is therefore no longer approved for these patients, regardless of cause of admission.
- No increase has been observed in the rate of AKI in surgical patients or in patients with haemorrhagic shock treated with HES.
- Administration of HES as a second line fluid solution reduces the overall volume of fluid administered to patients.
- The European Medicines Agency suggests that HES is optional as a second line fluid therapy following crystalloids in surgical patients, provided they are not septic or critically ill. This statement requires validation with additional RCTs.
- The data regarding gelatins or albumin in patients at risk of AKI is too sparse to draw meaningful conclusions.

#### Balanced versus unbalanced fluids

The clinical benefit of balanced-fluid resuscitation on renal function remains controversial [14]. A single center trial that compared chloride-liberal (saline, 4% gelatin, 4% albumin) to chloride-restrictive (lactated crystalloid, balanced crystalloid, 20% albumin) fluid administration in a nonselective cohort of 1500 ICU patients reported more renal dysfunction in the chloride-liberal group [13]. However, these findings must be interpreted with caution; the difference observed between the groups may have resulted not only from the dose of chloride administered but also from other potentially beneficial measures implemented only in the study group [13]. The large double-blind, cluster-randomized, double cross-over trial, compared 0.9% Saline versus Plasma-Lyte 148 for ICU fluid therapy (SPLIT) in 2300 hypovolemic patients [51]. No difference was found in the incidence of AKI, RRT between the 2 groups. However, both study and control groups received less fluids than expected; only 2655 ± 3052 and 2554 ± 2120 ml of study fluids were administered respectively during the 5-day study period. Moreover in the SPLIT trial, the patients were not severely ill and plasma chloride levels were not measured. A meta-analysis of critically ill and surgical patients showed no difference in the rates of mortality and RRT with balanced solutions when compared to unbalanced solutions [108]. However, meta-analyses on this topic are limited by large heterogeneities in case mix, fluid volumes and duration of exposure, underpowering, imprecision, and more.

In 2018, two large-scale randomized studies comparing balanced crystalloids versus saline were published, one in critically ill, and one in non critically ill patients [109, 110]. Among the 13,347 non-critically ill patients treated in the emergency department, there was no difference in hospital free days [110]. The trial comparing balanced crystalloids (Ringer’s solution or plasma-Lyte) to saline in 15,802 critically ill adults showed that the administration of balanced solutions resulted in lower rates of the composite outcome sought (death from any cause, new renal-replacement therapy, or persistent renal dysfunction) [109].

In practice, the systematic use of balanced solutions is not recommended in patients who are not critically ill yet and require low volume resuscitation. Experimental data and large observational studies support potential deleterious renal effects of unbalanced solutions related to severe hyperchloremia. The above mentioned large randomized trial in critically ill patients concluded that the use of balanced solution resulted in less use of renal replacement therapy, less persistent renal dysfunction, and higher survival [109]. A strategy favouring the use of balanced fluids in severe ICU patients requiring high fluid volume resuscitation is recommended [111, 112].

**Summary statements:**

- If a large volume of fluid is likely to be required for resuscitation, especially in septic patients, balanced fluid solutions should be selected as these may reduce the likelihood of AKI.
- Despite controversial data, balanced solutions for fluid resuscitation can be favoured even in with small amount of fluids as they may reduce the incidence of persistent renal dysfunction and the use of RRT.
- NaCl 0.9% remains useful for patients with hypochloremic alkalosis

### Future directions

In many patients stabilization of the systemic hemodynamic condition is not immediately accompanied by improvement in microcirculatory parameters. This situation may persist for hours or days, indicating long-lasting tissue ischemia [113]. Ongoing microcirculatory derangement is associated with increased morbidity and mortality, even when global hemodynamics are compensated [114]. Studies incorporating data on the effect of various fluids on the microcirculation are needed [115]. Dark-field microscopy, a new technique for measuring microcirculation, may offer important information regarding the microcirculatory changes occurring during administration of various fluids in specific disease conditions [116].

## Conclusions

Intravenous fluids are drugs and should be prescribed as such. Among the available fluids, crystalloids have the highest benefit/risk ratio and, should generally be prescribed first. For critically ill patients or when large amount of fluids is expected to be infused, balanced solutions should be preferred because of their favourable effects on patient outcomes, including kidney function. The preferred solution for non critically ill patients or low volume resuscitation is less clear. However, given the availability of balanced solutions and their low cost, they could be considered for all patients. The role of albumin remains a matter of debate, but there is indirect evidence that albumin may favourably affect the outcomes of patients with septic shock. The indications and effects of gelatins remain unclear for critically ill patients. The role of dextrans in this patient population should probably remain marginal until more data is forthcoming.

## Additional files

Additional file 1: Output of the systematic search. Description of data: Excel table reporting the output of the systematic search (XLSX 485 kb)

Additional file 2: Most relevant studies on fluids in critically ill patients discussed in the main text. Description of data: Table reporting relevant studies on fluids in critically ill patients retrieved by the systematic search (DOCX 151 kb)

## Abbreviations

AKI: Acute kidney injury
ARDS: Acute respiratory distress syndrome
CI: Cardiac index
CO: Cardiac output
CVP: Central venous pressure
EGDT: Early-goal directed therapy
GFR: Glomerular filtration rate
HES: Hydroxyethyl-starch
ICU: Intensive Care Unit
MFG: Modified fluid gelatine
NGAL: Neutrophil gelatinase-associated lipocain
PLR: Passive leg raising
RBF: Renal blood flow
RCT: Randomized controlled trial
SBP: Systolic blood pressure
SOFA: Sequential Organ Failure Assessment
SV: Stroke volume
TBI: Traumatic brain injury
