# Supplementary material for: Choice of fluids in critically ill patients
Source: BMC Anesthesiol. 2018 Dec 22;18:200. doi: 10.1186/s12871-018-0669-3 (PMC6303886; doi:10.1186/s12871-018-0669-3)
Supplement: Supplementary file 2 — Most relevant studies on fluids in critically ill patients discussed in the main text. Description of data: Table reporting relevant studies on fluids in critically ill patients retrieved by the systematic search (DOCX 151 kb) [file 12871_2018_669_MOESM2_ESM.docx]

| **First author, year of publication (reference number)** | **Study design**  **(number of patients)** | **Comparison** | **Population** | **First goal** |
| --- | --- | --- | --- | --- |
| Bickell et al. 1998 [1] | Comparative, non randomised (n=598) | Immediate vs. Delayed fluid resuscitation | Hypotensive trauma with torso penetrating injury | Hospital mortality |
| Schreiber et al. 2015 [2] | Randomised clinical trial (n=192) | Controlled resuscitation vs. Standard resuscitation | Out-of-hospital hypotensive trauma | Early crystalloid volume |
| Busuito et al. 2014 [3] | Subgroup analysis of randomised clinical trial (n=46) | Low FFP/RBC vs. high FFP/RBC  (non colloidal balanced solution vs. human serum albumin) | Trauma patients requiring transfusion | Fluid intake |
| Cooper et al. 2013 [4] | Post hoc analysis of SAFE study (n=460) | Albumin vs. Saline | ICU patients with traumatic brain injury | Mean change in intracranial pressure from randomisation to day 14 |
| Roquilly et al. 2013 [5] | Two-arm, randomised, double-blind, pilot controlled trial (n=42) | Isotonic balanced solutions vs. Isotonic chloride solutions | Severe traumatic brain injury or subarachnoid haemorrhage | Hyperchloraemic metabolic acidosis at 48 h |
| Young et al. 2015 [6] | Randomised, double-blind, parallel-group trial (n=65) | 0.9% NaCl vs. Plasma-Lyte A | Trauma patients requiring blood transfusion, intubation or operation | Base excess from 0 to 24 hours |
| Bulger et al. 2008 [7] | Double-blind, randomised clinical trial (n=209) | 250 mL of 7.5% hypertonic saline and 6% dextran 70 vs. lactated Ringer solution | Blunt trauma patients with prehospital hypotension | Survival without acute respiratory distress syndrome at 28 days |
| Wang et al. 2014 [8] | Meta-analysis (6 studies and 1254 participants) | Hypertonic saline vs. Isotonic saline | Trauma patients | Mortality, blood pressure, fluid requirement, serum sodium |
| Han et al. 2015 [9] | Double-blind randomised clinical trial (n=294) | 3% hypertonic saline solution vs. 7.5% hypertonic saline solutions vs. Standard fluid resuscitation | Severe trauma patients with pre-hospital hypotension | Time to mean arterial pressure restoration |
| Annane et al. 2013 [10] | Randomised clinical trial (n=2857) | Colloids vs. crystalloids | ICU patients including trauma and sepsis | 30-day mortality |
| Myburgh et al. 2012 [11] | Randomised clinical trial patients=532) | 6% hydroxyethylstarch vs. Saline | ICU patients including trauma and sepsis | 90-day mortality |
| Guidet et al 2012 [12] | Randomized clinical trial | 6% hydroxy ethyl starch vs 0.9% saline | Severe sepsis patients | Amount of fluid to correct hemodynamic abnormalities |
| Raghunathan et al  2014 [13] | Retrospective cohort | Balanced vs no-balanced fluids | Septic patients | In-hospital mortality |
| Finfer et al 2004 [14] | Randomized clinical trial | Albumin vs 0.9% saline | Septic, trauma, and ARDS patients | 28-day mortality |
| Wiedemann et al. 2006 [15] | Randomized clinical trial (n=1000) | Conservative vs. liberal fluid strategy | Patients receiving mechanical ventilation, with PaO_2/_FiO_2_ < 300, bilateral infiltrates on chest radiography, no left atrial hypertension | Death from any cause at 60 days |
| Martin et al. 2005  [16] | Randomized double-blind placebo controlled trial (n=40) | Furosemide with albumin vs. furosemide with placebo titrated to fluid loss and serum protein concentration | Patients with acute lung injury/ARDS and serum total protein concentration <6 g/dl | Change in oxygenation over a 24-hour period |
| Mikkelsen et al. 2012 ^(65)^ | Adjunct study of a randomized controlled trial (FACTT trial) (n=122) | Conservative vs. liberal fluid strategy | Survivors from acute lung injury/ARDS, enrolled in the FACTT trial | Neuropsychological function at 2 and 12 months post-hospital discharge |
| Uhlig et al 2014 [17] | Systematic review and meta-analysis | Albumin versus crystalloid | Patients with ARDS | Respiratory mechanics, gas exchange, hospital mortality |
| Silversides et al 2017 [18]^(68)^ | Systematic review and meta-analysis (2051) | Conservative fluid strategy or deresuscitation vs liberal fluid strategy or standard care | Patients with ARDS, sepsis, SIRS | Mortality, ventilator-free days |
| Semler MW et al. 2018 [19] | Comparative, cluster-randomised multiple crossover, unblinded, multicenter (n=15802) | Saline crystalloid vs. balanced crystalloid resuscitation | Critically ill adults incuding sepsis and trauma | Major adverse kidney events within 30 days (composite including in-hospital mortality, new RRT & persistent renal dysfunction |
| Perner A et al. 2012 [20] | Randomised, blinded multicentre parallel group (n=798) | 6% HES vs. Ringer Acetate resuscitation | Critically ill adults with severe sepsis | Survival |
| Sakr Y et al. 2017 [21] | Observational cohort study (audit in 84 countries) (n=1808) | Impact of fluid overload | ICU critically ill adults with sepsis | 28 day in-hospital death |
| Kim IY et al. 2017 [22] | Retrospective data collection (n=341) | Impact of fluid overload | ICU critically ill adults with AKI receiving CRRT | 30-day mortality |
| Legrand M et al. 2013 [23] | Retrospective single centre study(n=137) | Impact of systemic hemodynamic parameters | Critically ill patients with septic AKI | Development of new or persistent AKI |
| Lipcsey M al. 2015 [24] | Prospective observational (101) | Impact of the primary fluid bolus for hypotensive resuscitation | Emergency department patients with infection-associated hypotension | Systemic hemodynamic parameters |
| Zarychanski R et al. 2013 [25] | Systematic review & meta-analysis (n=10880) | Hydroxyethylstarch vs crystalloid, albumin or gelatin acute resuscitation | Critically ill adults | Mortality,j real failure, RRT replacement |
| Gattas DJ et al.  2013 [26] | Systematic review & meta-analysis (10391) | Hydroxyethylstarch 6% vs crystalloid or colloid | Critically ill patients & perioperative patients | Mortality & need of RRT |
| Serpa Neto A et al.  2014 [27] | Systematic review & meta-analysis (4624) | HES vs crystalloid resuscitation | Critically ill septic adults | Incidence of AKI & use of RRT |
| He B et al.  2015 [28] | Systematic review & meta-analysis (6064) | HES vs other fluids resuscitation | Critically ill non-septic patients | Mortality, RRT incidence |
| Van der Linden P et al. 2013 [29] | Systematic review & Meta-analysis (4529) | Tetrastarch vs other fluids (crystalloid, gelatin) resuscitation | Surgical patients during surgey | Safety (mortality, coagulation, transfusion, AKI) |
| Martin C et al.  2013 [30] | Systematic review & Meta-analysis (1230) | HES vs other fluid resuscitation | Surgical patients | Occurrence or renal dysfunction |
| Bayer O et al.  2013 [31] | Prospective observational cohort study (6478) | Synthetic colloids (tetraspan & gelatin) resuscitation | Cardiac surgery patients (cardiopulmonary bypass) | Need of RRT & fluid requirement |
| Kashy BK et al.  2014 [32] | Prospective observational cohort study (44176) | HES + crystalloid vs crystalloid alone resuscitation | Non cardiac surgical patients | AKI risk |
| Joosten A et al.  2018 [33] | Randomised, double-blind controlled trial (160) | Intraoperative balanced crystalloid vs colloid using closed loop system administration | Major abdominal surgical patients | Postoperative morbidity survey score at Day 2 |
| Caironi P et al.  2014 [34] | Randomised, milticenter, open label trial (1818) | 20% albumin + crystalloid vs crystalloid alone resuscutation | Criticallly ill adults with severe sepsis | Death at day 28 |
| Yunos M et al.  2015 [35] | 2-year extended study of a previous before-after study (2994) | Liberal chloride rich-fluids (control) vs restricted chloride rich-fluid (interventional) resuscitation | Consecutive critically ill adults | Incidence of AKI |
| Yunos M et al.  2012 [36] | Prospective, open-label, before-after pilot study (1533) | Standard fluids (control) vs restricted chloride rich-fluid (interventional) resuscitation | Critically ill adults including septic patients (7.2 % vs 10.0 %) | Increase creatinine from baseline to peak & incidence of AKI |
| Wilcox CS  1983 [37] | Experimental, comparative prospective (25 dogs) | Intrarenal infusion of hypertonic NaCl vs NaHCO3 vs Na acetate vs dextrose vs NaH4 acetate | Experimental study | Renal blood flow & glomerular filtration rate |
| Young P et al.  2015 [6] | Double-blind, double crossover, cluster, randomised (2278) | Buffered crystalloid vs saline crystalloid resuscitation | Critically ill adults | Proportion of AKI |
| Kawano-Dourado L et al. 2017 [38] | Systematic review & meta-analysis (n=3170) | HES vs crystalloid resuscitation | Critically & perioperative adults | Morality rate & RRT Use |
| Self WH et al.  2018 [39] | Comparative, multiple cross over, unblinded, single-center (n=13347) | Balanced fluid vs. non-balanced fluids resuscitation | Non critically ill adults in the emergency department | Hospital free-days to day 28 (composite in-hospital & LOS at D28) |

**Additional file 2:** Most relevant studies on fluids in critically ill patients retrieved by the systematic search

AKI: acute kidney injury; ARDS: Adult respiratory distress syndrome; ES: hydroxyethylstarch; ICU: intensive care unit; LOS: length of stay; RRT: renal replacement therapy

1. Bickell WH, Wall MJJ, Pepe PE, Martin RR, Ginger VF, Allen MK, et al. Immediate versus delayed fluid resuscitation for hypotensive patients with penetrating torso injuries. N Engl J Med. 1994;331:1105–9.

2. Schreiber MA, Meier EN, Tisherman SA, Kerby JD, Newgard CD, Brasel K, et al. A controlled resuscitation strategy is feasible and safe in hypotensive trauma patients: results of a prospective randomized pilot trial. J Trauma Acute Care Surg. 2015;78:687–95–discussion695–7.

3. Busuito CM, Ledgerwood AM, Lucas CE. Colloid with high fresh frozen plasma/red blood cell resuscitation does not reduce postoperative fluid needs. J Trauma Acute Care Surg. 2014;76:1008–12.

4. Cooper DJ, Myburgh J, Heritier S, Finfer S, Bellomo R, Billot L, et al. Albumin resuscitation for traumatic brain injury: is intracranial hypertension the cause of increased mortality? J Neurotrauma. 2013;30:512–8.

5. Roquilly A, Loutrel O, Cinotti R, Rosenczweig E, Flet L, Mahe PJ, et al. Balanced versus chloride-rich solutions for fluid resuscitation in brain-injured patients: a randomised double-blind pilot study. Crit Care. 2013;17:R77.

6. Young P, Bailey M, Beasley R, Henderson S, Mackle D, McArthur C, et al. Effect of a Buffered Crystalloid Solution vs Saline on Acute Kidney Injury Among Patients in the Intensive Care Unit: The SPLIT Randomized Clinical Trial. JAMA. 2015;314:1701–10.

7. Bulger EM, Jurkovich GJ, Nathens AB, Copass MK, Hanson S, Cooper C, et al. Hypertonic resuscitation of hypovolemic shock after blunt trauma: a randomized controlled trial. Arch Surg. 2008;143:139–48–discussion149.

8. Wang J-W, Li J-P, Song Y-L, Tan K, Wang Y, Li T, et al. Hypertonic saline in the traumatic hypovolemic shock: meta-analysis. J Surg Res. 2014;191:448–54.

9. Han J, Ren H-Q, Zhao Q-B, Wu Y-L, Qiao Z-Y. Comparison of 3% and 7.5% Hypertonic Saline in Resuscitation After Traumatic Hypovolemic Shock. Shock. 2015;43:244–9.

10. Annane D, Siami S, Jaber S, Martin C, Elatrous S, Declere AD, et al. Effects of fluid resuscitation with colloids vs crystalloids on mortality in critically ill patients presenting with hypovolemic shock: the CRISTAL randomized trial. JAMA. 2013;310:1809–17.

11. Myburgh JA, Finfer S, Bellomo R, Billot L, Cass A, Gattas D, et al. Hydroxyethyl starch or saline for fluid resuscitation in intensive care. N Engl J Med. 2012;367:1901–11.

12. Guidet B, Martinet O, Boulain T, Philippart F, Poussel JF, Maizel J, et al. Assessment of hemodynamic efficacy and safety of 6% hydroxyethylstarch 130/0.4 vs. 0.9% NaCl fluid replacement in patients with severe sepsis: the CRYSTMAS study. Crit Care. 2012;16:R94.

13. Raghunathan K, Shaw A, Nathanson B, Sturmer T, Brookhart A, Stefan MS, et al. Association between the choice of IV crystalloid and in-hospital mortality among critically ill adults with sepsis*. Crit Care Med. 2014;42:1585–91.

14. Finfer S, Bellomo R, Boyce N, French J, Myburgh J, Norton R. A comparison of albumin and saline for fluid resuscitation in the intensive care unit. N Engl J Med. 2004;350:2247–56.

15. Wiedemann HP, Wheeler AP, Bernard GR, Thompson BT, Hayden D, deBoisblanc B, et al. Comparison of two fluid-management strategies in acute lung injury. N Engl J Med. 2006;354:2564–75.

16. Martin GS, Moss M, Wheeler AP, Mealer M, Morris JA, Bernard GR. A randomized, controlled trial of furosemide with or without albumin in hypoproteinemic patients with acute lung injury. Crit Care Med. 2005;33:1681–7.

17. Uhlig C, Silva PL, Deckert S, Schmitt J, de Abreu MG. Albumin versus crystalloid solutions in patients with the acute respiratory distress syndrome: a systematic review and meta-analysis. Critical Care. BioMed Central; 2014;18:R10–0.

18. Silversides JA, Major E, Ferguson AJ, Mann EE, McAuley DF, Marshall JC, et al. Conservative fluid management or deresuscitation for patients with sepsis or acute respiratory distress syndrome following the resuscitation phase of critical illness: a systematic review and meta-analysis. Intensive Care Med. 2017;43:155–70.

19. Semler MW, Self WH, Wanderer JP, Ehrenfeld JM, Wang L, Byrne DW, et al. Balanced Crystalloids versus Saline in Critically Ill Adults. N Engl J Med. 2018;378:829–39.

20. Perner A, Haase N, Guttormsen AB, Tenhunen J, Klemenzson G, Aneman A, et al. Hydroxyethyl starch 130/0.42 versus Ringer's acetate in severe sepsis. N Engl J Med. 2012;367:124–34.

21. Sakr Y, Rubatto Birri PN, Kotfis K, Nanchal R, Shah B, Kluge S, et al. Higher Fluid Balance Increases the Risk of Death From Sepsis: Results From a Large International Audit*. Crit Care Med. 2017;45.

22. Kim IY, Kim JH, Lee DW, Lee SB, Rhee H, Seong EY, et al. Fluid overload and survival in critically ill patients with acute kidney injury receiving continuous renal replacement therapy. Burdmann EA, editor. PLoS One. Public Library of Science; 2017;12:e0172137.

23. Legrand M, Dupuis C, Simon C, Gayat E, Mateo J, Lukaszewicz A-C, et al. Association between systemic hemodynamics and septic acute kidney injury in critically ill patients: a retrospective observational study. Crit Care. BioMed Central; 2013;17:R278.

24. Lipcsey M, Chiong J, Subiakto I, Kaufman M, Schneider AG, Bellomo R. Primary fluid bolus therapy for infection-associated hypotension in the emergency department. Critical Care and Resuscitation. The Australasian Medical Publishing Company; 2015;17:6.

25. Zarychanski R, Abou-Setta AM, Turgeon AF, Houston BL, McIntyre L, Marshall JC, et al. Association of hydroxyethyl starch administration with mortality and acute kidney injury in critically ill patients requiring volume resuscitation: a systematic review and meta-analysis. JAMA. American Medical Association; 2013;309:678–88.

26. Gattas DJ, Dan A, Myburgh J, Billot L, Lo S, Finfer S. Fluid resuscitation with 6 % hydroxyethyl starch (130/0.4 and 130/0.42) in acutely ill patients: systematic review of effects on mortality and treatment with renal replacement therapy. Intensive Care Med. 2013;39:558–68.

27. Serpa Neto A, Veelo DP, Peireira VGM, de Assuncao MSC, Manetta JA, Esposito DC, et al. Fluid resuscitation with hydroxyethyl starches in patients with sepsis is associated with an increased incidence of acute kidney injury and use of renal replacement therapy: a systematic review and meta-analysis of the literature. J Crit Care. 2014;29:185.e1–7.

28. He B, Xu B, Xu X, Li L, Ren R, Chen Z, et al. Hydroxyethyl starch versus other fluids for non-septic patients in the intensive care unit: a meta-analysis of randomized controlled trials. Critical Care. London: BioMed Central; 2015;19:92.

29. Van der Linden P, James M, Mythen M, Weiskopf RB. Safety of modern starches used during surgery. Anesth Analg. 2013;116:35–48.

30. Martin C, Jacob M, Vicaut E, Guidet B, Van Aken H, Kurz A. Effect of waxy maize-derived hydroxyethyl starch 130/0.4 on renal function in surgical patients. Anesthesiology. 2013;118:387–94.

31. Bayer O, Schwarzkopf D, Doenst T, Cook D, Kabisch B, Schelenz C, et al. Perioperative fluid therapy with tetrastarch and gelatin in cardiac surgery--a prospective sequential analysis*. Crit Care Med. 2013;41:2532–42.

32. Kashy BK, Podolyak A, Makarova N, Dalton JE, Sessler DI, Kurz A. Effect of hydroxyethyl starch on postoperative kidney function in patients having noncardiac surgery. Anesthesiology. 2014;121:730–9.

33. Joosten A, Delaporte A, Ickx B, Touihri K, Stany I, Barvais L, et al. Crystalloid versus Colloid for Intraoperative Goal-directed Fluid Therapy Using a Closed-loop System: A Randomized, Double-blinded, Controlled Trial in Major Abdominal Surgery. Anesthesiology. 2018;128:55–66.

34. Caironi P, Tognoni G, Masson S, Fumagalli R, Pesenti A, Romero M, et al. Albumin replacement in patients with severe sepsis or septic shock. N Engl J Med. 2014;370:1412–21.

35. Yunos NM, Bellomo R, Glassford N, Sutcliffe H, Lam Q, Bailey M. Chloride-liberal vs. chloride-restrictive intravenous fluid administration and acute kidney injury: an extended analysis. Intensive Care Med. 2015;41:257–64.

36. Yunos NM, Bellomo R, Hegarty C, Story D, Ho L, Bailey M. Association between a chloride-liberal vs chloride-restrictive intravenous fluid administration strategy and kidney injury in critically ill adults. JAMA. 2012;308:1566–72.

37. Wilcox CS. Regulation of renal blood flow by plasma chloride. J Clin Invest. 1983;71:726–35.

38. Kawano-Dourado L, Zampieri FG, Azevedo LCP, Correa TD, Figueiro M, Semler MW, et al. Low- Versus High-Chloride Content Intravenous Solutions for Critically Ill and Perioperative Adult Patients: A Systematic Review and Meta-analysis. Anesth Analg. 2018;126:513–21.

39. Self WH, Semler MW, Wanderer JP, Wang L, Byrne DW, Collins SP, et al. Balanced Crystalloids versus Saline in Noncritically Ill Adults. N Engl J Med. 2018;378:819–28.
